# Peripheral Nerve Imaging Aids in the Diagnosis of Immune-Mediated Neuropathies—A Case Series

**DOI:** 10.3390/diagnostics10080535

**Published:** 2020-07-30

**Authors:** Marc Dörner, Frank Schreiber, Heike Stephanik, Claus Tempelmann, Natalie Winter, Jan-Hendrik Stahl, Julia Wittlinger, Sophia Willikens, Magdalena Kramer, Hans-Jochen Heinze, Stefan Vielhaber, Thomas Schelle, Alexander Grimm, Stefanie Schreiber

**Affiliations:** 1 Center for Neurology, Tuebingen University Hospital and Hertie-Institute for Clinical Brain Research, Eberhard Karls University Tuebingen, 72076 Tuebingen, Germany; Natalie.winter@med.uni-tuebingen.de (N.W.); Jan-hendrik.stahl@med.uni-tuebingen.de (J.-H.S.); julia.wittlinger@med.uni-tuebingen.de (J.W.); Sophia.willikens@med.uni-tuebingen.de (S.W.); magdalena.kramer@med.uni-tuebingen.de (M.K.); alexander.grimm@med.uni-tuebingen.de (A.G.); 2 Department of Neurology, Otto-von-Guericke University, 39120 Magdeburg, Germany; frank.schreiber@dzne.de (F.S.); heike.stephanik@med.ovgu.de (H.S.); claus.tempelmann@med.ovgu.de (C.T.); hans-jochen.heinze@med.ovgu.de (H.-J.H.); stefan.vielhaber@med.ovgu.de (S.V.); stefanie.schreiber@med.ovgu.de (S.S.); 3 German Center for Neurodegenerative Diseases (DZNE) within the Helmholtz Association, 39120 Magdeburg, Germany; 4 Center for Behavioural Brain Sciences (CBBS), 39120 Magdeburg, Germany; 5 Leibniz Institue for Neurobiology (LIN), 39120 Magdeburg, Germany; 6 Department of Neurology, Städtisches Klinikum Dessau, 06847 Dessau, Germany; thomas.schelle@klinikum-dessau.de

**Keywords:** peripheral nerve imaging, nerve ultrasound, magnetic resonance neurography, immune-mediated neuropathies, amyotrophic lateral sclerosis

## Abstract

Background: Diagnosis of immune-mediated neuropathies and their differentiation from amyotrophic lateral sclerosis (ALS) can be challenging, especially at early disease stages. Accurate diagnosis is, however, important due to the different prognosis and available treatment options. We present one patient with a left-sided dorsal flexor paresis and initial suspicion of ALS and another with multifocal sensory deficits. In both, peripheral nerve imaging was the key for diagnosis. Methods: We performed high-resolution nerve ultrasound (HRUS) and 7T or 3T magnetic resonance neurography (MRN). Results: In both patients, HRUS revealed mild to severe, segmental or inhomogeneous, nerve enlargement at multiple sites, as well as an area increase of isolated fascicles. MRN depicted T2 hyperintense nerves with additional contrast-enhancement. Discussion: Peripheral nerve imaging was compatible with the respective diagnosis of an immune-mediated neuropathy, i.e., multifocal motor neuropathy (MMN) in patient 1 and multifocal acquired demyelinating sensory and motor neuropathy (MADSAM) in patient 2. Peripheral nerve imaging, especially HRUS, should play an important role in the diagnostic work-up for immune-mediated neuropathies and their differentiation from ALS.

## 1. Introduction

Multifocal motor neuropathy (MMN) and multifocal acquired demyelinating sensory and motor neuropathy (MADSAM), also called Lewis–Sumner syndrome, are chronic immune-mediated neuropathies caused by an autoimmune response to peripheral nerves [1]. Both diseases are characterized by slowly progressive asymmetric focal limb weakness and distinguished by additional multifocal sensory deficits in the latter, which is also defined as an atypical variant of chronic inflammatory demyelinating polyneuropathy (CIDP) [2]. Especially at the early stages a distinction between these two diseases and amyotrophic lateral sclerosis (ALS) might be difficult, considering that each of them, MMN, MADSAM and ALS, can show symptoms such as progressive focal weakness, fasciculations, muscle cramps, atrophy in the affected muscles and decreased tendon reflexes [3].

Whereas ALS is a devastating neurodegenerative disease with a poor prognosis, MMN and MADSAM have a rather good outcome if treated immediately with intravenous immunoglobulins (IVIG; MMN and MADSAM), corticosteroids or plasmapheresis (MADSAM) [4]. Thus, an accurate diagnosis and understanding of these diseases is of utmost importance.

So far, MMN, MADSAM and ALS diagnoses have been commonly based on clinical and electrophysiological grounds [5,6,7,8]. However, when applying those criteria, 4 to 10% of all initial ALS diagnoses are incorrect [9,10]. Through follow-up, around 20% of them will be later re-diagnosed to suffer from MMN, which is one of the most common ALS mimics [10].

Recently, high-resolution nerve ultrasound (HRUS) has been considered potentially useful to additionally aid in the differential diagnosis between immune-mediated neuropathies such as MMN and MADSAM and motor neuron diseases such as ALS [11,12,13]. The feasibility of other nerve imaging methods, such as magnetic resonance neurography (MRN), to reliably differ between these diagnoses still needs more in-depth investigation [14].

We here present one patient with an initial suspicion of ALS and another with multifocal sensory deficits where peripheral nerve imaging was the key for the respective diagnosis of an immune-mediated neuropathy.

### 1.1. Case Presentation

#### 1.1.1. Patient 1

A 54-year old man presented with a mild flaccid dorsal flexor paresis of the left foot and big toe, fasciculations of the upper arms and upper legs and an increased tendency for muscle cramps, which had lasted for two months. He had a decreased Achilles and normal masseter but otherwise symmetric vivid tendon reflexes (patellar, triceps and biceps reflex), a flexor plantar reflex and a positive Trömner’s reflex on both sides. There were no additional neurologic symptoms (pathological laughter/crying, dysphagia, dysarthria, sensory loss) or signs (split hand syndrome) and no muscle atrophy. Initial electroneurography (ENG) depicted reduced compound muscle action potential (CMAP) amplitudes (left tibial nerve), but normal motor conduction velocity (MCV) and no conduction block (CB) or sensory impairment (Table 1). Electromyography (EMG) revealed ongoing denervation (i.e., abnormal spontaneous activity (positive sharp waves, fibrillation potentials)) and re-innervation (i.e., motor unit potentials with increased amplitudes, durations and number of phases) of the bilateral tibialis anterior and the left interosseus dorsalis I (hand) muscles. Cerebrospinal fluid (CSF) showed an albuminocytologic dissociation (protein concentration: 110 mg/dL (15–45 mg/dL [15]); cell count: 4 cells/µL (5)) and increased neurofilament light chain (NFL) levels (5539 pg/mL (612–2616 pg/mL [16])). Remaining CSF diagnostic work-up variables and IgM anti-ganglioside GM1 antibodies were in the normal range. Serum creatine kinase was slightly increased (543 U/L (< 172 U/L [17])). The patient met the Awaji criteria for possible ALS [5,6]. Alternative diagnoses of infectious, vascular and toxic origin were excluded via comprehensive blood testing (including thyroid and endocrinologic dysfunction) and cerebral and spinal cord MR imaging (MRI). However, ALS diagnosis was doubted according to high CSF protein, which is a supportive criterion for an immune-mediated neuropathy, e.g., MMN [8]. To move forward in the differentiation between ALS and MMN at that early disease stage, we performed HRUS and 7T MRN.

#### 1.1.2. Patient 2

The second patient, a 51-year old man, reported a fluctuating (digiti IV/V) or permanent (digiti I–III) progressive numbness without pain of his left hand, starting five years ago. There was a further intermittent fine motor dysfunction of his left hand (digiti I–III) and a numbness of his right toes, forefoot and sole. Concurrent diagnoses (spinal cord involvement, nerve compression syndromes) had already been excluded. Muscle tendon reflexes were normal, pyramidal signs were negative and there was no palsy. ENG revealed reduced MCV and sensory nerve action potential of the left median nerve as well as a partial motor CB of the left median and right peroneal nerve; there were no abnormalities of the remaining nerves, distal motor latency or F-wave latency (Table 1). As the results thus far had not been conclusive, the patient requested further strengthening of the suspected diagnosis. He declined invasive diagnostics, such as lumbar puncture, and demanded non-invasive options instead. We suggested to perform HRUS and MRN.

## 2. Materials and Methods

### 2.1. High-Resolution Nerve Ultrasound

HRUS was performed using an eL18-4 18 MHz broadband ultrasound probe (Philips Medical Systems Affiniti 70 G (Bothell, WA, USA). Examination of patient 1 took place two (and eight and eighteen) months after symptom onset, while patient 2 underwent ultrasound six years after symptom onset. In patient 1, bilateral median and ulnar nerves from the wrist to the upper arm and distal tibial nerves were examined. In patient 2, nerve sonography was performed according to the ultrasound pattern sum score (UPSS) comprising the examination of bilateral sensorimotor nerves (median, ulnar, peroneal and tibial nerves; ultrasound pattern score A (UPSA)), cervical roots (C5, C6) and vagal nerves (UPSB) and sural nerves (UPSC) at predefined sites, respectively [18]. Cross-sectional nerve areas within the hyperechoic epineural rim (CSA, for all nerves), diameter (for C5, C6) and area of enlarged appearing single fascicles (measured inside the hyperechoic rim corresponding to CSA assessment) were measured [18,19,20,21]. Nerve vascularization was assessed in a semiquantitative manner applying power Doppler (grade 0: no vascularity; grade 1: 1 or 2 focal color-encoded spots; grade 2: 1 linear color-encoded line or > 2 focal color-encoded spots; grade 3: > 1 linear color-encoded line [22]).

### 2.2. MR Neurography

In patient 1, 7T MRN of the bilateral distal tibial nerve was conducted using a Siemens Healthineers MAGNETOM scanner (Erlangen/Germany) with a 28-channel knee coil applying a high-resolution 2-dimensional T2-weighted turbo spin echo sequence with fat-saturation. In patient 2, 3 T MRN of the left upper arm median nerve and the left brachial plexus was performed using a Siemens Healthineers MAGNETOM Skyra 3T scanner (Erlangen/Germany) with an 18-channel body coil utilizing the same T2-weighted imaging and, additionally, a contrast-enhanced (gadolinium) T1-weighted sequence. In patient 1, MRN was conducted two months after symptom onset and in patient 2 it was performed six years after symptom onset.

## 3. Results

### 3.1. Patient 1

**Figure 1:**
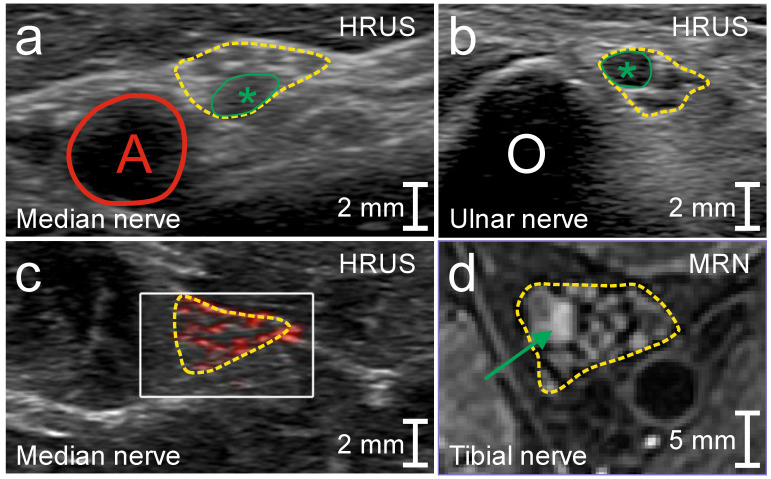
Peripheral nerve imaging in patient 1 (MMN). High-resolution nerve ultrasound (HRUS) demonstrated mild inhomogeneous enlargement of the cross-sectional nerve area (CSA, yellow in (**a**–**d**); (**a**) right upper arm median nerve, CSA 17 mm^2^; (**b**) left ulnar nerve at the cubital tunnel, CSA 13 mm^2^) and isolated prominent (green asterisk in (**a**), 3.7 mm^2^) or enlarged fascicle area (green asterisk in (**b**), 4.1 mm^2^) as well as increased nerve vascularizaton with multiple focal color-encoded spots (grade 2; within the yellow border in (**c**)). 7T magnetic resonance neurography (MRN) depicted isolated fascicles that were T2 hyperintense and enlarged (green arrow in (**d**); left tibial nerve). A, brachial artery; O, olecranon.

HRUS showed mild and inhomogeneous enlargement of the bilateral median (up to 1.6-fold), ulnar (up to 1.5-fold) and tibial nerves (up to 1.5-fold) (Table 2; Figure 1a,b). An isolated prominent fascicle (3.7 mm^2^) was found in the right upper arm median nerve (Figure 1a) and isolated fascicle enlargement became obvious in the left ulnar nerve at the cubital tunnel (4.1 mm^2^; ≤2.8 mm^2^; [21]) (Figure 1b). Nerve vascularization was slightly increased (grade 2). 7T MRN revealed T2 hyperintensity and enlargement of isolated fascicles in the bilateral tibial nerve (Figure 1c).

### 3.2. Patient 2

**Figure 2:**
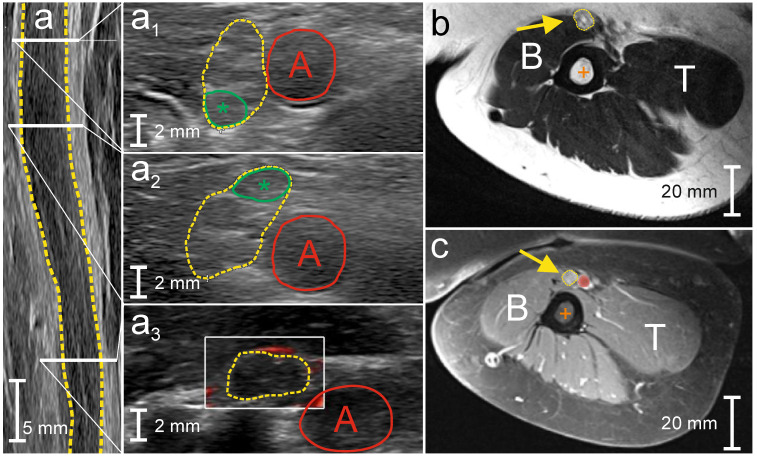
Peripheral nerve imaging in patient 2 (MADSAM). High-resolution nerve ultrasound (HRUS) demonstrated severe, asymmetric, fusiform and segmental enlargement of the cross-sectional nerve area (CSA, yellow in (**a**–**a_3_**)) of the left upper arm median nerve ((**a_1_**) CSA 23.7 mm^2^; (**a_2_**) 32.1 mm^2^; (**a_3_**) 12 mm^2^) and isolated fascicle area enlargement (green asterisk in (**a_1_**) and (**a_2_**), area in (**a_1_**) 5.6 mm^2^; in (**a_2_**) 6.1 mm^2^). Nerve vascularization was not increased (grade 0; within the yellow border in (**a_3_**)) 3T magnetic resonance neurography (MRN) of the left upper arm median nerve displayed isolated fascicles with contrast-enhancement (yellow arrow in (**b**) contrast-enhanced (gadolinium) T1-weighted sequence) and T2 hyperintensity (yellow arrow in (**c**) T2-weighted turbo spin echo sequence with fat saturation). Red dot, brachial artery; A, brachial artery; orange cross, humerus; B, biceps brachii muscle; T, triceps brachii muscle. In **a**, the long-axis HRUS of the left upper arm median nerve view is given, while (**a_1_**–**a_3_**) show the corresponding short-axis view.

HRUS displayed severe, up to 2.7-fold fusiform and segmental asymmetric CSA enlargement of the left upper arm median nerve (Table 2, Figure 2a) and mild inhomogeneous, up to 1.7-fold asymmetric CSA enlargement of the right tibial nerve (Table 2). Multiple enlarged fascicles were depicted in the left upper arm median nerve (area up to 6.1 mm^2^; ≤4.8 mm^2^; [21]; Figure 2a_2_). The remaining UPSS/A/B/C measuring sites did not show any abnormalities; nerve vascularization was not increased (grade 0). 3T MRN revealed upper arm median nerve T2 hyperintensity and mild contrast-enhancement (Figure 2b,c).

## 4. Discussion

In patient 1 and patient 2, HRUS and MRN were potentially compatible with an immune-mediated neuropathy, indicated by mild to severe, segmental or inhomogeneous, asymmetric nerve CSA and fascicle enlargement at some sites, increased nerve vascularization and T2 hyperintense and contrast-enhancing fascicles [13,25]. These findings, especially the segmental and asymmetric nerve enlargement, have been reported in several HRUS und MRN studies taking account of MMN and MADSAM patients [13,26,27,28]. We were thus convinced to diagnose MMN (patient 1) and MADSAM (patient 2) based on peripheral nerve imaging*,* even though not all of the diagnostic findings were supportive of these diseases when applying common criteria (patient 1: pyramidal signs, motor involvement of only one nerve, no CB, IgM anti-ganglioside GM1 antibodies not elevated; patient 2: normal tendon reflexes in affected limbs, missing CSF protein and nerve biopsy results). Brisk tendon reflexes can be identified in up to 20% of MMN cases [9]. Thus, these findings in patient 1 did not rule out MMN.

We initiated IVIG therapy 2 months (patient 1: 30 g/d over five days per session) and 6 years (patient 2: 90 g/d over two days per session) after symptom onset. Within the first four months after IVIG onset (patient 1), muscle strength of the left dorsal flexor remained on a constant level (Medical Research Council scale (MRC): 4/5 before IVIG, 4 + / 5 after IVIG onset). Sixteen months (patient 1) after IVIG onset, the patient displayed further disease progression, now having a bilateral dorsal flexor paresis of his feet (MRC: 0–1/5). A progressive deterioration of symptoms is even typical under IVIG therapy [29]. Patient 2 showed steady results in the neurological examination without further disease progression six months after IVIG onset. Electrodiagnosic follow-up in patient 1 (11 and 16 months after IVIG initiation, 13 and 18 months after symptom onset) revealed further CMAP and, now, MCV reduction of the bilateral tibial and the right peroneal nerve (together with a new-diagnosed probable CB 13 months after symptom onset) (Table 1).

Systematic HRUS studies reported mild (up to 1.4-fold) nerve enlargement in around 20% of all ALS patients as well [11,30]; in a single ALS case nerve area increase was even 1.8-fold [31]. ALS patients with some nerve enlargement also show a higher CSF albumin/serum albumin ratio indicative of a blood-nerve barrier breakdown and might include more male cases with longer disease duration and positive superoxide dismutase 1 mutation carrier status [30]. Underlying inflammation of the peripheral nervous system (PNS) might contribute to HRUS nerve enlargement, suggesting some pathophysiological overlap between PNS involvement in ALS and immune-mediated neuropathies [32]. Certainly, possible CSA enlargement in ALS is not that pronounced as in MMN or MADSAM and is rather homogeneous and symmetric [29,33]. Fascicular enlargement and increased nerve vascularization have, however, thus far not been detected in ALS patients [33,34], but further studies are needed. Additionally, several ALS patients showed fascicular T2 hyperintensities, especially those in whom initially an immune-mediated neuropathy had been suspected [34,35,36]. Further, patient 1 exhibited (slightly) increased CSF NfL levels, which can be detected in both MMN and ALS [16,37]. Bearing in mind these overlapping findings, peripheral nerve imaging should always be used in the context of a larger spectrum of diagnostic modalities. If nerve ultrasound is added to conventional diagnostics, the detection rate of immune-mediated neuropathies is thereby improved by 20% [38]. Yet some patients with immune-mediated neuropathies show normal CSA values [13]. In these cases, HRUS might be not sufficient for differential diagnosis. As there are also ALS patients having nerve CSA enlargement, combining HRUS and MRN can help differentiate these diseases (see above). Another advantage of combining both technologies is that they can compensate each other’s limitations (HRUS: displaying distal nerves, nerve vascularization, long-distance nerve segments; MRN: showing blood-nerve barrier breakdown through T2 sequences, proximal nerve segments). Combining HRUS and MRN and applying new imaging markers might thereby be a promising approach to achieve even more conclusive and diagnosis-guiding results [14,39,40]. Of course, future systematic studies have to prove if combining both technologies will improve diagnostic accuracy [40]. Still, combining clinical examination, nerve conduction studies and HRUS is sufficient for the diagnosis of immune-mediated neuropathies in some cases and MRN is hence not necessary [13].

The follow-up HRUS in patient 1 six months after IVIG onset (eight months after symptom onset) displayed smaller nerve CSA values compared with the initial HRUS. CSA values were still enlarged, but the enlargement pattern changed from mild inhomogeneous to mild homogeneous. However, sixteen months after IVIG onset (eighteen months after symptom onset) a further follow-up HRUS revealed again increased nerve CSA values, indicating ongoing disease activity (Table 2). Literature about the monitoring of treatment response with HRUS in MMN is, however, sparse. There is one case, where IVIG treatment led to the normalization of CSA nerve values [41], while another study reported fascicle area regression under IVIG therapy (but also in therapy naive MMN patients) [42]. By contrast, Leeuw et al. [31] demonstrated a patient with multifocal nerve enlargement and initial suspicion of an immune-mediated neuropathy. After a clinical follow-up examination and generalized CSA decrease, diagnosis was changed to ALS. In line with this, Schreiber et al. [20] described a progressive CSA decline in ALS patients over a time span of 15 months, albeit this study did not take into account whether there was initial nerve enlargement. However, further systematic studies are warranted to understand the underlying nature of longitudinal CSA changes.

There are some limitations of the diagnostic work-up of the cases. Additional diffusion tensor or muscle MRI could have further aided in the differentiation between MMN or ALS [43,44]. However, due to the lack of standardized protocols for nerve and muscle imaging (especially for MRI), the differentiation between ALS mimics and ALS remains challenging, even in face of the availability of multiple imaging-based measures. Furthermore, Oudeman et al. [45] found out that intraobserver and interobserver agreements vary considerably regarding MRN imaging. Hypertrophy as well as increased signal intensity can be even frequently found in healthy controls and not only in patients with suspected immune-mediated neuropathies. Considering these results, there might be a possible lack of significance of hyperintensity. Imaging-based differential diagnosis between its mimics and ALS is thus still an individual decision of the caring physician/neurologist [14]. In patient 1, CB might have been found at diagnostic work-up through more extended electrodiagnostics. In patient 2, invasive diagnostics such as lumbar puncture and nerve biopsy did not take place, which are supportive criteria in the diagnosis of CIDP [7]. Another limitation is that nerve conduction studies were not performed according to the EFNS protocol. However, both cases picture “everyday” difficulties of physicians’ lives filled with time pressure and missing diagnostics.

## 5. Conclusions

In both patients, peripheral nerve imaging played an important role in the diagnosis. Further studies are needed to prove the clinical benefit of MRN in the diagnosis of immune-mediated neuropathies and ALS. HRUS has already proven to be potentially useful in the diagnostic work-up and monitoring of disease activity of these disorders [46]. Still, clinical examination and electrodiagnostic studies remain the diagnostic cornerstones, but HRUS could serve as a “door opener” if diagnostic findings are inconclusive. In the future, diagnostic criteria for immune-mediated neuropathies and ALS might include HRUS, if not as an obligate, then at least as a supportive criterion.

## Figures and Tables

**Table 1:** Standard nerve conduction studies in relation to symptom onset.

Patient	1									2		
Time Point	M 2			M 13			M 18			Y 5		
Nerve (Stimulation)	DML (ms)	CMAP (mV)	MCV (m/s)	DML (ms)	CMAP (mV)	MCV (m/s)	DML (ms)	CMAP (mV)	MCV (m/s)	DML (ms)	CMAP (mV)	MCV (m/s)
Motor												
**Peroneal** (ankle) R				4.3 (5.0)	**1.0** (2.1)		**6.4** (5.0)	**0.4** (2.1)		3.9 (5.0)	4.2 (2.1)	
L	4.7 (5.0)	2.3 (2.1)										
(Fibula head) R					**1.0** (2.1)	**36.4** (41)		**0.5** (2.1)	**36** (41)		2.7 ^b^ (2.1)	44.5 (41)
L		2.1 (2.1)	49.3 (41)									
**Tibial** (ankle) R							4.7 (5.1)	**0.6** (2.9)				
L	**5.2** (5.1)	**2.0** (2.9)		**6.2** (5.1)	**0.1** (2.9)							
(popliteal fossa) R								**0.5** (2.9)	**39.8** (40)			
L		**2.0** (2.9)	49.9 (40)		***0.2** (2.9)*	**34.7** (40)						
**Median** (wrist) R	2.9 (4.5)	7.9 (2.9)		2.8 (4.5)	*9.9 (2.9)*		2.9 (4.5)	9.4 (2.9)				
L							3.1 (4.5)	6.2 (2.9)		3.0 (4.5)	8.7 (2.9)	
(elbow) R		7.1 (2.9)	59.1 (47)		6.4 ^a^ (2.9)	54.3 (47)		8.7 (2.9)	56.2 (47)			
(axilla) L								5.5 (2.9)	56 (47)		5.0 ^c^ (2.9)	**33.1 ^d^** (47)
**Ulnar** (wrist) R	2.5 (3.5)	10.9 (2.5)		2.7 (3.5)	11.0 (2.5)		2.5 (3.5)	9.3 (2.5)				
L							2.8 (3.5)	9 (2.5)		2.6 (3.5)	7.7 (2.5)	
(elbow) R		10.8 (2.5)	67.9 (48)		10.3 (2.5)	69.8 (48)		8.9 (2.5)	66.5 (48)			
L								6.9 (2.5)	66.2 (48)		5.6 (2.5)	64.4 (48)
**Sensory**		**SNAP (µV)**	**SCV (m/s)**		**SNAP (µV)**	**SCV (m/s)**		**SNAP (µV)**	**SCV (m/s)**		**SNAP (µV)**	**SCV (m/s)**
**Sural** (ankle) R											3.7 (3.5)	45.1 (38)
L		21.8 (3.5)	48.8 (38)		6.0 (3.5)	41.8 (38)		14.8 (3.5)	48.5 (38)			
**Median** (dig. II) R					5.7 (2)	52.7 (44)		8.3 (2)	52.4 (44)		8.5 (2)	55.2 (44)
L		9.4 (2)	60.7 (44)		10.8 (2)	56.8 (44)		6.3 (2)	58.3 (44)		**0.9 (2)**	46.6 (44)
**Ulnar** (dig. V) R					4.8 (2)	60.6 (43)		5.2 (2)	58.1 (43)			
L		4.8 (2)	59.9 (43)					7.4 (2)	55 (43)		2.0 (2)	48.0 (43)

Note: Pathological values are marked bold. Reference values are given in brackets. Abbreviations: M = month; Y = year; R = right; L = left; DML = distal motor latency; CMAP = compound muscle action potential; MCV = motor conduction velocity; SNAP = sensory nerve action potential; SCV = sensory conduction velocity. ^a^ probable CB: negative peak CMAP area reduction > 35% (wrist to elbow). ^b^ probable CB: negative peak CMAP area reduction > 35% (ankle to fibula head). ^c^ probable CB: negative peak CMAP area reduction > 42% (wrist to axilla). ^d^ > 29% below lower limit of normal values.

**Table 2:** Nerve ultrasound cross-sectional area (CSA) values in mm^2^ in relation to symptom onset.

Patient		1			2
Time Point		M 2	M 8	M 18	Y 6
**Median nerve wrist**	R	**17** (13 ^*^)	**13** (13 ^*^)	**13** (13 ^*^)	8 (13 ^*^)
	L	**13** (13 ^*^)	**16** (13 ^*^)	**14** (13 ^*^)	11 (13 ^*^)
**Median nerve forearm**	R	**12** (10)	**11** (10)	**13** (10)	7 (10)
	L	**16** (10)	**11** (10)	**12** (10)	7 (10)
**Median nerve elbow**	R				9–11 (12.5)
	L				**17** (12.5)
**Median nerve upper arm**	R	**17** (12)	**14** (12)	**14** (12)	9 (12)
	L	**18** (12)	**17** (12)	**18** (12)	**12–32** (12)
**Ulnar nerve wrist**	R			7 (8 ^**^)	
	L			**9** (8 ^**^)	7 (8 ^**^)
**Ulnar nerve forearm**	R	**11** (8.5)	**9** (8.5)	**12** (8.5)	4 (8.5)
	L	**13** (8.5)	**10** (8.5)	**12** (8.5)	6 (8.5)
**Ulnar nerve cubital tunnel**	R	**9** (9–10 ^**^)			
	L	**13** (9–10 ^**^)			6 (9–10 ^**^)
**Ulnar nerve upper arm**	R			**15** (9.5)	
	L			**16** (9.5)	7 (9.5)
**Tibial nerve distal**	R	**21** (14)	**20** (14)		**24** (14)
	L	**20** (14)	**23** (14)		**15** (14)
**Tibial nerve proximal**	R				**35** (33)
	L				28 (33)
**Peroneal nerve proximal**	R				5 (11.5)
	L				5 (11.5)

Note: Pathological values are marked bold. Reference values are given in brackets [18], ^*^ [23], ^**^ [24]. Abbreviations: M = month; Y = year; R = right; L = left.
